# Creatine monohydrate increases skeletal muscle microvascular blood flow and promotes lipid mobilization in postmenopausal women

**DOI:** 10.1080/15502783.2025.2533650

**Published:** 2025-08-21

**Authors:** Paul A. Baker, Sequoia D. Ernst, John C. DeCaro, Riley K. Hart, Mostafa M. Ali, Joesph C. Watso, Michael J. Ormsbee, Justin D. La Favor, Abbie E. Smith-Ryan, Robert C. Hickner

**Affiliations:** a Applied Physiology Laboratory, Department of Exercise and Sport Science, University of North Carolina at Chapel Hill, Chapel Hill, NC; b Department of Health, Nutrition, and Food Sciences, Florida State University, Tallahassee, FL, United States; c Institute of Sports Sciences and Medicine, Florida State University, Tallahassee, Florida, United States; d School of Health Sciences, Discipline of Biokinetics, Exercise and Leisure Sciences, University of KwaZulu-Natal, Durban, South Africa; e Human Movement Science Curriculum, Department of Health Sciences, University of North Carolina at Chapel Hill, Chapel Hill, NC; f Department of Nutrition, Gillings School of Public Health, University of North Carolina at Chapel Hill Chapel Hill, NC

**Keywords:** Creatine, microvascular circulation, lipid mobilization, postmenopause, reactive oxygen species, cardiovascular physiological phenomena

## Abstract

**Background:**

Postmenopausal females have an elevated cardiovascular disease (CVD) risk, induced partly by reduced skeletal muscle microvascular blood flow (SMBF), elevated reactive oxygen (ROS), and impaired meal metabolism (glycerol, glucose, lactate). Evidence suggests that creatine monohydrate (CrM), holds promise for reducing CVD risk via improvements in blood flow and ROS reductions; however, data are limited. The current pilot study investigated the impact of CrM on SMBF, ROS concentrations, and markers of meal metabolism at rest and following the consumption of a high carbohydrate (HC) meal (a potent stimulator of ROS), in postmenopausal females.

**Methods:**

Six postmenopausal females (66 ± 7 yrs, 30.9 ± 3.59 kg/m^2^) were enrolled in this randomized, double-blind, crossover study. Participants completed two randomized study arms: CrM (20g/day) and placebo (PL, maltodextrin, 20g/day), each for five days, separated by a four-week washout period. At PRE- and POST-supplementation visits, a microdialysis probe was inserted into the gastrocnemius muscle to measure SMBF (ethanol outflow/inflow ratio (o:i)), [H_2_O_2_] (an index of ROS), and dialysate contents. Following a 45min equilibrium period, participants rested for one hour and then consumed a HC meal (35% of daily energy requirements; ~80% carbohydrates). Dialysate samples were collected every 20 minutes and data is combined (1hr basal + 4hr post-prandial). ClinicalTrials.gov ID #NCT06018480.

**Results:**

Five days of CrM increased SMBF (mean±SD; PRE CrM:0.71±0.11o:i, POST CrM: 0.61±0.10o:i; Visit*Treatment p<0.0001) compared to five days of PL (PRE PL: 0.65±0.19o:i, POST PL: 0.63±0.14o:i; Visit*Treatment p=0.44). CrM had no effect on [H_2_O_2_] (PRE CrM:1.24±0.73µM, POST CrM: 1.21±0.19µM; Visit*Treatment p=0.99). CrM increased dialysate glycerol (0.67±0.32μmol/L, 0.75±0.25μmol/L; Visit*Treatment p=0.04), with no change in dialysate glucose (Visit*Treatment p=0.49), and a non-significant decrease in dialysate lactate (Visit*Treatment p=0.06).

**Conclusions:**

Five days of CrM increased SMBF and dialysate glycerol, suggesting enhanced blood flow and lipid mobilization that may support a reduction in CVD risk in postmenopausal females.

